# Surface ruptures and off-fault deformation of the October 2016 central Italy earthquakes from DInSAR data

**DOI:** 10.1038/s41598-022-07068-9

**Published:** 2022-02-24

**Authors:** Filippo Carboni, Massimiliano Porreca, Emanuela Valerio, Manzo Mariarosaria, Claudio De Luca, Salvatore Azzaro, Maurizio Ercoli, Massimiliano R. Barchi

**Affiliations:** 1 Dipartimento di Fisica e Geologia, Centro InteRUniversitario per l’analisi Sismotettonica Tridimensionale (CRUST), Università degli studi di Perugia, 06123 Perugia, Italy; 2 Istituto per il Rilevamento Elettromagnetico dell’Ambiente, IREA-CNR, Naples, Italy

**Keywords:** Natural hazards, Structural geology, Geophysics, Seismology, Tectonics

## Abstract

Large magnitude earthquakes produce complex surface deformations, which are typically mapped by field geologists within the months following the mainshock. We present detailed maps of the surface deformation pattern produced by the M. Vettore Fault System during the October 2016 earthquakes in central Italy, derived from ALOS-2 SAR data, via DInSAR technique. On these maps, we trace a set of cross-sections to analyse the coseismic vertical displacement, essential to identify both surface fault ruptures and off-fault deformations. At a local scale, we identify a large number of surface ruptures, in agreement with those observed in the field. At a larger scale, the inferred coseismic deformation shows a typical long-wavelength convex curvature of the subsiding block, not directly recognizable in the field. The detection of deformation patterns from DInSAR technique can furnish important constraints on the activated fault segments, their spatial distribution and interaction soon after the seismic events. Thanks to the large availability of satellite SAR acquisitions, the proposed methodological approach can be potentially applied to worldwide earthquakes (according to the environmental characteristics of the sensed scene) to provide a wider and faster picture of surface ruptures. Thus, the derived information can be crucial for emergency management by civil protection and helpful to drive and support the geological field surveys during an ongoing seismic crisis.

## Introduction

Earthquakes can produce a wide spectrum of surface deformations associated with the main events or between inter-seismic periods^1,2^. The largest events (M_w_ > 5) can trigger deformations such as surface ruptures related to the activation of main active faults and/or other deformations induced by seismic shaking (e.g., landslides, creeping, sinkholes).

In the last three decades, remote sensing acquisitions such as Differential Synthetic Aperture Radar Interferometry (DInSAR), Lidar differencing, optical imagery, and GPS^3,4^ have been implemented to reach an impressively high accuracy, useful for detecting detailed surface deformations. In particular, DInSAR is one of the most powerful and reliable techniques to provide a snapshot of the coseismic deformations^5^ generated by earthquakes occurred worldwide within different complex geological and morphological settings^6^.

**Figure 1:**
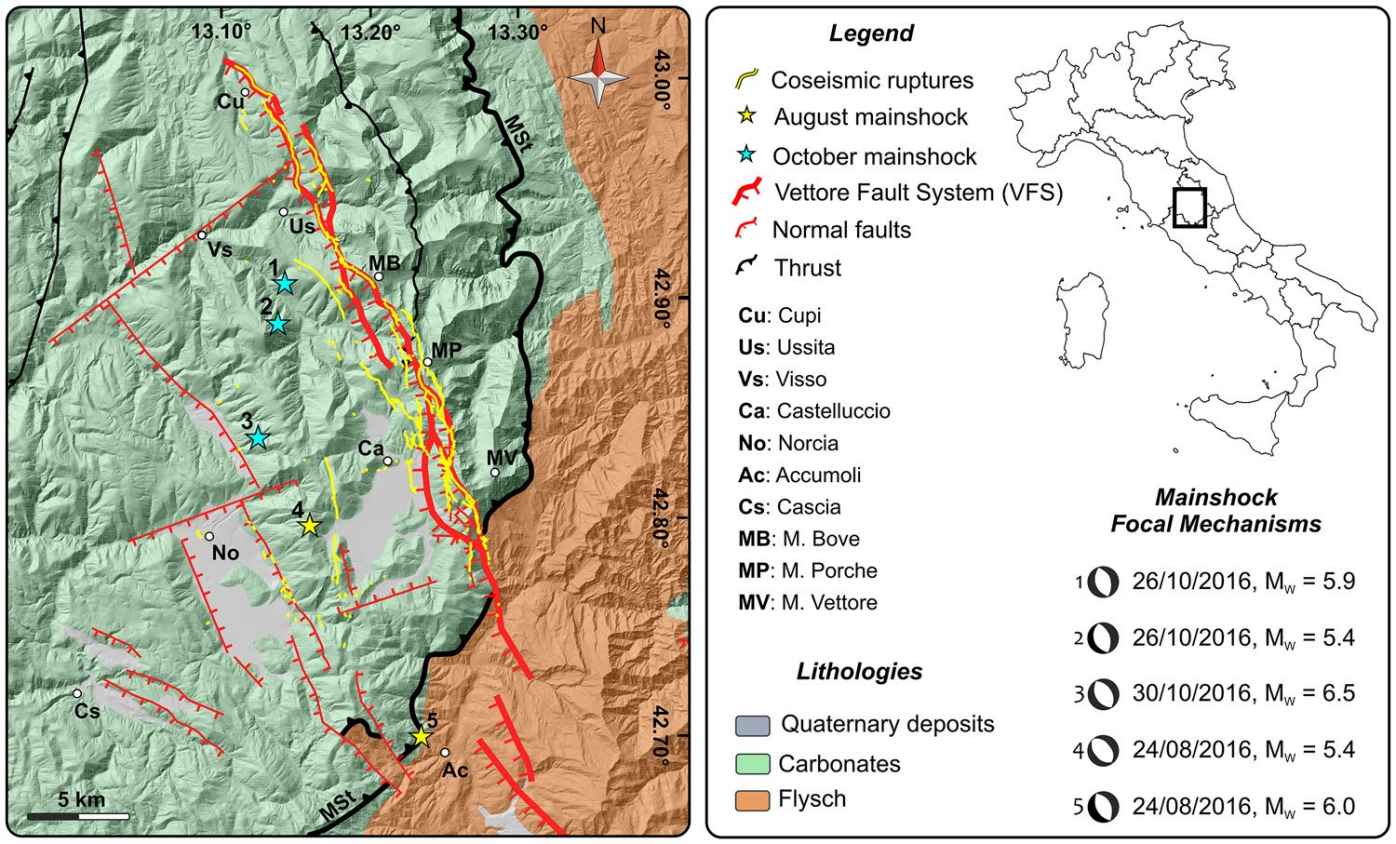
Geological map. Geological scheme of the study area (modified after^22^) and coseismic ruptures observed in the field along with the main shocks of the 2016–2017 earthquake sequence.

In 2016, a long earthquake sequence affected a tectonically complex region of the Apennines in central Italy (Fig. 1), producing impressive surface ruptures due to the activation of the SW-dipping extensional M. Vettore Fault System (VFS). Most of these ruptures are attributed to the 24 August Mw 6.0 and 30 October Mw 6.5 main-shocks and have been investigated by several groups of field geologists soon after the earthquakes^7,8^. The results of the field surveys indicate a complex distribution of the ruptures reflecting the geological complexity of the epicentral area, where a succession of both carbonate and siliciclastic rocks crops out^7,8^.

The struck area has been also investigated by DInSAR technique, aiming at studying the rock volumes affected by deformation, the faulting mechanics, the source geometry and local to large-scale surface deformation^4,9–15^. However, these data have not yet been used to investigate in detail the single rupture’s locations associated with the largest mainshocks. Here, we use SAR data acquired from the ALOS-2 system to generate line-of-sight (LOS) displacement maps over ascending and descending orbits to retrieve the corresponding vertical and horizontal displacement fields (VDM and HDM, respectively) affecting the investigated area.

We propose a new approach aimed at a detailed identification and analysis of all the single surface ruptures within the entire deformation field produced by the earthquakes. In this way, we are able to analyse, at the same time, regional and local scale deformations at an accuracy never experienced before. The results of this work show the potential of our approach in analysing DInSAR data to readily trace coseismic deformations, even if located in remote and complex areas. Such displacement maps can therefore be considered an added valuable information to support field activities during seismic emergencies.

## Seismotectonic setting

The area affected by the 2016 seismic events is localized in a tectonically complex region of the central Apennines (Fig. 1), whose structural evolution is characterized by a first Late Miocene—Early Pliocene compressional phase, followed by Late Pliocene—Quaternary extension^16,17^. The compressional phase has been controlled by the M. Sibillini thrust^18^ (MSt, Fig. 1) which marks the tectonic boundary between the Mesozoic—Paleogene carbonate sequence at its hangingwall, and the Late Miocene—Early Pliocene flysch at its footwall (Fig. 1).

The N–S trending compressional structures are displaced by later NNW-SSE trending normal faults, even if their cross-cutting relationships are still debated^19–21^. The VFS (Fig. 1), characterized by several fault segments^22^, is the main extensional structure. The seismic sequence, which ruptured the entire VFS, started with the 24 August 2016 Mw 6.0 and Mw 5.4 events, followed, two months later, by the 26 October Mw 5.9 and the 30 October Mw 6.5 events^23^; this work is focused on the deformation associated with the October earthquakes.

The activation of the VFS during the largest events is recorded by surface ruptures developed along the major SW-dipping fault segments and minor synthetic and antithetic structures depicting a complex deformation pattern^8,24–27^.

## Data and methods

We use SAR images collected from the ALOS-2 system between the 24 August 2016 (excluding the 24 August main-shock) and the 6 September 2017, coupled to generate four interferometric pairs (Table 1 and Fig. S1). Data from the 24 August 2016 (acquired immediately after the 24 August Accumoli event), the 2 November 2016 and the 6 September 2017 have been collected over ascending orbit, while data from the 31 August 2016, 9 November 2016 and 24 May 2017, over descending orbit.

**Table 1:** ALOS-2 coseismic interferometric pairs exploited for the DInSAR analysis.

Sensor	InSAR pair	Orbit	Track	Wavelength (cm)	Perpendicular baseline (m)	Look angle (deg)
ALOS-2	24/08/2016–02/11/2016	ASC	197	24.2	99	36.6
ALOS-2	31/08/2016–09/11/2016	DESC	92	24.2	56	36.8
ALOS-2	24/08/2016–06/09/2017	ASC	197	24.2	127	36.6
ALOS-2	31/08/2016–24/05/2017	DESC	92	24.2	15	36.8

In this work, the exploited DInSAR measurements have been retrieved by using the Parallel Small BAseline Subsets (P-SBAS) algorithm^28^. These SAR acquisitions have been exploited to compute the differential interferograms and the corresponding radar line-of-sight (LOS) displacement maps. We remark that the processing is carried out at the ALOS-2 full spatial resolution up to interferogram generation. At this step, to produce the differential interferograms, a multi-look operation is implemented to increase the signal–noise ratio. In addition, to remove the topographic phase contribution, the SRTM-1arcsec Digital Elevation Model (DEM) has been used. The corresponding displacement maps have a ~ 30 m-pixel resolution and are estimated through an appropriate phase unwrapping operation^29^. Subsequently, we combine these displacement maps^30,31^ to obtain vertical and horizontal (East–West) coseismic displacement components maps. More in detail, we combine the displacement maps with shorter timespan over ascending (24/08/2016–02/11/2016) and descending orbits (31/08/2016–09/11/2016) to retrieve the VDM1 and HDM1 maps (Fig. S2a,b). Instead, the displacement maps with longer timespan over ascending (24/08/2016–06/09/2017) and descending orbits (31/08/2016–24/05/2017) have been used to generate VDM2 and HDM2 maps (Fig. S2c,d).

The comparison between the two sets of displacement maps, allows us to better discern the actual zones affected by ground deformation from a possible influence of the atmospheric phase artefacts.

The interferometric pair has been imported into MOVE software (Petroleum Experts Ltd.) as a point cloud, to be subsequently interpolated. The interpolation has been performed by using two different algorithms, Delaunay Triangulation^32^ and Ordinary Kriging^33^, using suggested parameters, to verify their influence on the output surface. Since the two interpolations gave the same analytical result, we can assume that, with such a dense points cloud, the output surfaces are not affected by interpolation methods. We decided to use the surface created by Delaunay Triangulation for the analysis performed in this work.

We analyse the coseismic deformation patterns mainly on the VDMs since the studied earthquakes are mainly controlled by prevalent dip-slip movement^34^. In particular, we focus on the analysis of the VDM1, which involves the temporally shorter and less noisy interferometric pairs. Nevertheless, we also inspect the VDM2 (Figs. S2, S3), to better discriminate the zones actually affected by surface deformation from possible undesired phase artefacts (e.g., atmospheric phase delays, phase unwrapping errors).

**Figure 2:**
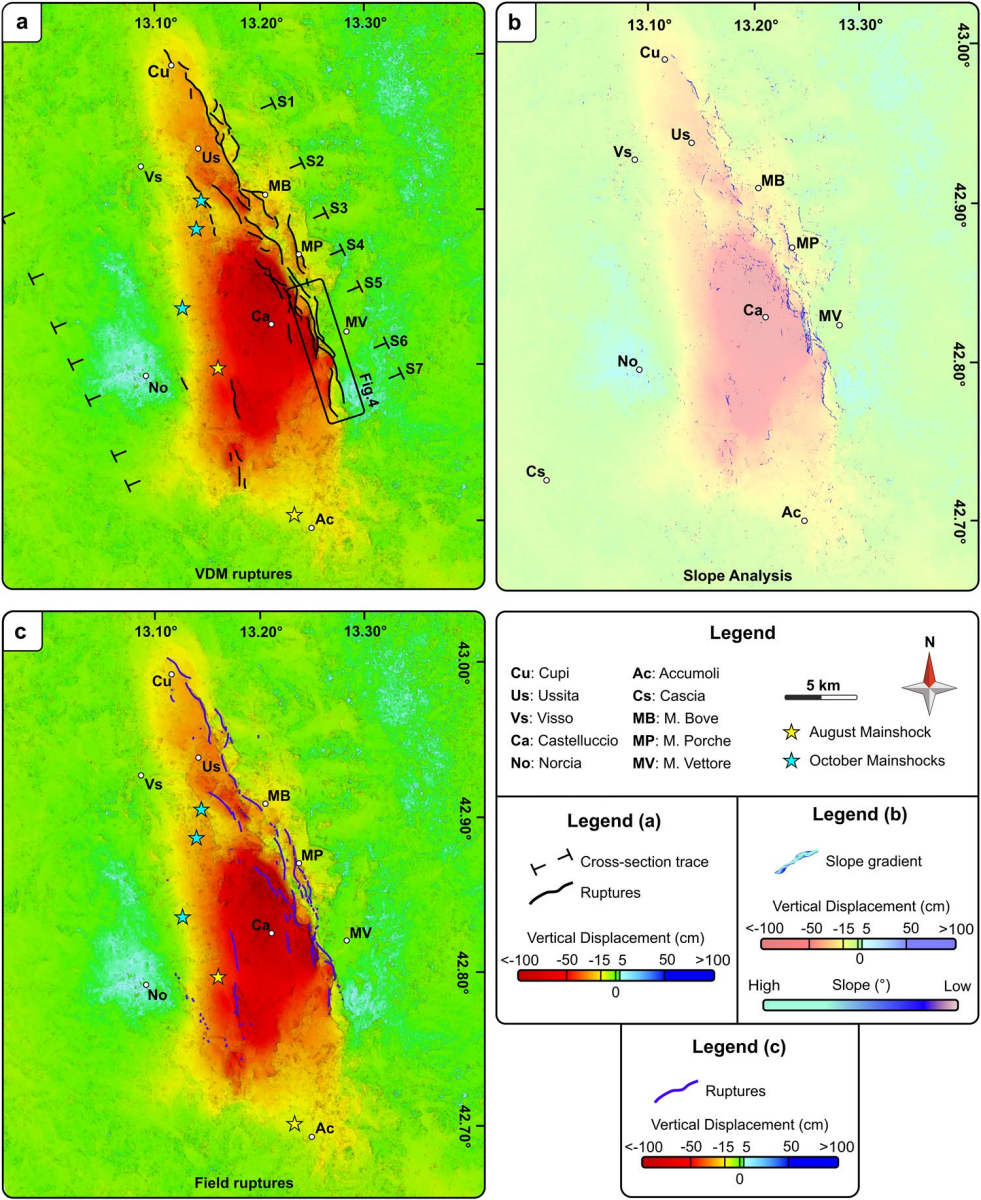
Comparison between DInSAR and field data. (**a**) Vertical displacement map (VDM1) with traces of the coseismic surface ruptures from this study and the cross-section traces (labelled S1 to S7). (**b**) Results of the slope analysis carried out on the whole studied area and superimposed on the VDM1. The blue zones correspond to steep vertical gradients of the VDM1 and are interpreted as coseismic ruptures. (**c**) Vertical displacement map (VDM1) with traces of the coseismic surface ruptures from the field surveys^8^ (purple lines).

**Figure 3:**
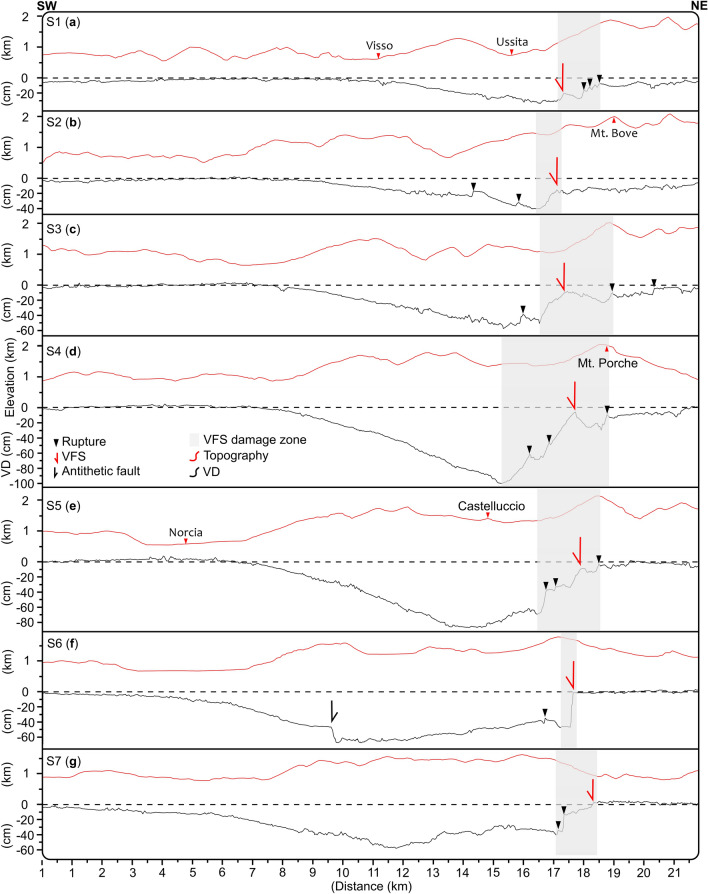
VDM1 cross-sections. Seven representative cross-sections showing the vertical displacement (VD, in black), and the topography (in red). The grey areas represent the zones affected by deformation close to the VFS. The VFS, the antithetic fault, the VD and the ruptures have been derived from the VDM1. Vertical exaggeration of the VD =  × 25.

Starting from the VDM1 (Fig. S2a), we investigate the surface ruptures and large-scale deformation tracing a set of 35 cross-sections covering the whole analyzed area. These transects are 1-km spaced and orthogonal to the mean strike of the VFS (Fig. 2a). In Fig. 3, we report a selection of seven sections, which are representative of the overall deformation pattern.

Due to the complexity of the deformed zone, we apply an integrated approach to trace the surface ruptures by means of the analysis of the displacement gradient on both the cross-sections and VDM1. On the cross-sections, we consider vertical displacements higher than 3 cm^14^, corresponding to about 1/8 of the exploited ALOS-2 system wavelength, which represents a realistic error range for the estimated coseismic displacements. On VDM1, we identify the steep displacement gradient computing the slope (see Fig. 2b and the raster file in the supplementary material) by using the QGIS software, that provides the rate of the vertical displacement (i.e., deformation gradient) ranging from 0° to 30°. This latter approach allows to automatically trace the surface ruptures. The length of each rupture is ensured by cross-checking the amounts of displacement on the sections and the lateral continuity of the slope values calculated on the VDM1. Finally, the retrieved ruptures (Fig. 2a) are subsequently compared and validated with those recognized from the field surveys^8^ (Fig. 2c).

## Results and discussion

The VDM1 shows a generalized subsidence of the hanging-wall block characterized by a peculiar triangular shape (Fig. 2), with a maximum value (~ − 101 cm) recorded north of Castelluccio di Norcia (dark red). A weak uplift (~ + 17 cm) is recorded in the Norcia area, to the west of the subsiding sector (light blue). The maximum displacement gradient, marked by the sharp contact between green to red patterns (Fig. 2) follows the complex trace of the seismogenic VFS.

The selected cross-sections (Fig. 3) show a southward increase of the subsidence, ranging from a maximum of 30–40 cm to the north (sections S1–S3), up to 85–100 cm in the central part of the subsiding area (sections S4, S5), which also corresponds to the area of maximum horizontal displacement (Fig. S4). While the VFS hanging-wall is characterized by a long-wavelength upward-convex curvature (Fig. 3), the VFS fault zone is characterized by sharp, well-localised vertical displacements. In the southern sector, the long-wavelength deformation is less evident and locally interrupted to the south by a steep vertical gradient, testifying the occurrence of an antithetic NE-dipping fault (Fig. 3f).

Besides considering the deformation caused by the October earthquakes, our data also include the post-seismic deformation following the 24 August earthquake. However, we assume that the small post-seismic deformation, estimated to ranging from 13.2 ± 1.4 to 35.5 ± 1.7 mm from 30 October 2016 to 6 January 2017^35^, is negligible (i.e., in the range of the error of the DInSAR dataset) when compared with the total amount of deformation.

Despite thousands of coseismic ruptures have been recognized in the field, for a cumulative length of ca. 88 km, we are able to recognize evidence of unclear and debated seismic ruptures recognized in the field. In detail, even if we identify a lower number of coseismic ruptures, these are characterized by higher lateral continuity, up to a cumulative length of ca. 100 km.

**Figure 4:**
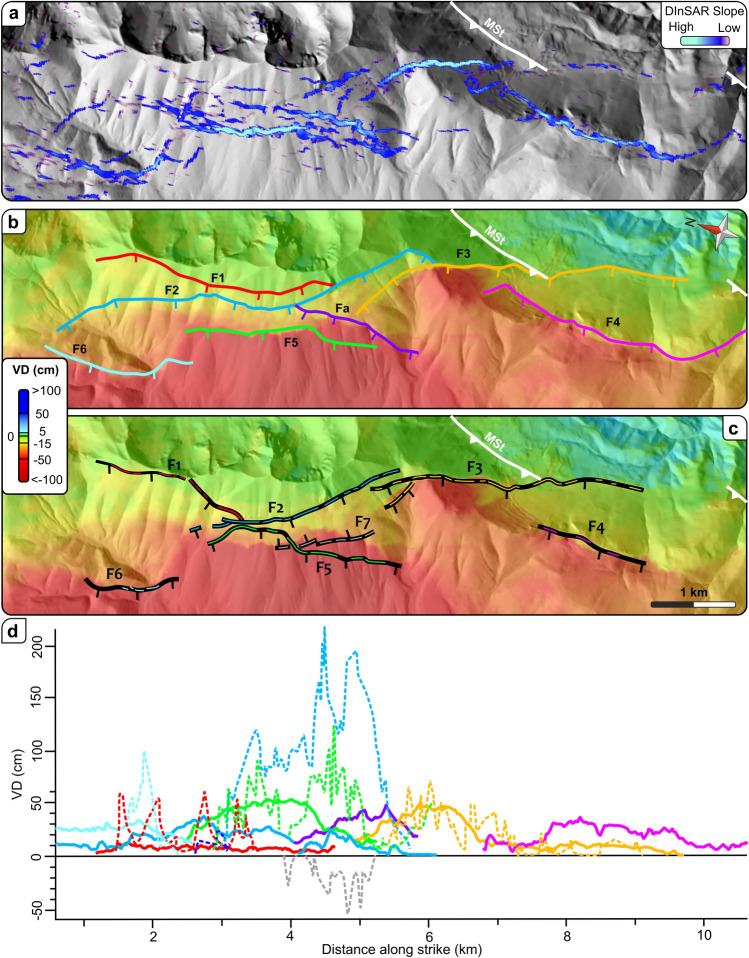
Comparison of field and DInSAR results of the M. Vettore area. (**a**) Slope distribution obtained from the VDM1, superimposed on the DEM. (**b)** Coseismic ruptures derived from the VDM1. (**c)** Coseismic ruptures mapped in the field^8^. The white lines mark the trace of the M. Sibillini Thrust (MSt). (**d**) Comparison of the vertical displacement (VD) accrued by single ruptures as observed in the field (dotted lines) and from the VDM1 (continuous lines).

We focus on the area south of M. Vettore (Fig. 4, inset in Fig. 2a) where, although high resolution kinematic field data are available, the activation of some fault segments and cross-cutting relationships are still debated. We thus report a comparison among the VDM1 slope analysis (Fig. 4a), the DInSAR- (Fig. 4b) and the field-derived surface ruptures^7,8^ (Fig. 4c). The results of the first two analyses allow us to further extend the lateral continuity of the coseismic ruptures, which are characterized by higher lengths than those observed in the field; this is strongly evident by comparing F1, F2, F3, F4 and F6 segments (Fig. 4b,c). In particular, we retrieve a ~ 5 km long rupture in the southern sector (F4 in Fig. 4b), which is longer than the SW-dipping fault segment previously mapped^7^. In the same sector, we are also able to confirm the southward continuity of the F3 segment, cross cutting the MSt (Fig. 4b); this segment is clearly visible in the field when cuts carbonate sequence, whereas is less clear in siliciclastic deposits^8,36^. In addition, we observe a minor correspondence in a restricted zone on the steep SW slope of Mt. Vettore, where some segments, characterized by different orientation, dip and displacements, are located in a zone affected by high decorrelation noise of the DInSAR data. Indeed, we recognize a SW-dipping segment (Fa in Fig. 4b), not observed in the field, located in the same area of an antithetic segment (F7 in Fig. 4c), which in turn we are not able to identify.

A quantitative comparison of DInSAR- and field-derived vertical displacement^8^ (Fig. 4d) reveals that our approach is particularly effective to constrain ruptures characterized by spatial vertical displacement up to 50–60 cm, which, in the field, show an unclear lateral continuity. For instance, we show that F3 is characterized by a high displacement with a peak of 50 cm in carbonate rocks at the MSt hangingwall, decreasing down to 10 cm in siliciclastic rocks at the MSt footwall.

The overall results highlight a good agreement in the spatial distribution of the DInSAR-derived and field ruptures; moreover, thanks to the VDM1, we are able to recognize evidence of debated coseismic ruptures recognized in the field. On the other hand, the performed quantitative analysis reveals that the vertical displacement values obtained by DInSAR data, are generally lower than those measured in the field (Fig. 4d). These discrepancies occur because the interferometric phase is strongly noisy close to ruptures as displacement exceed a single-phase cycle, which corresponds to half wavelength of the transmitted signal^37^.

**Figure 5:**
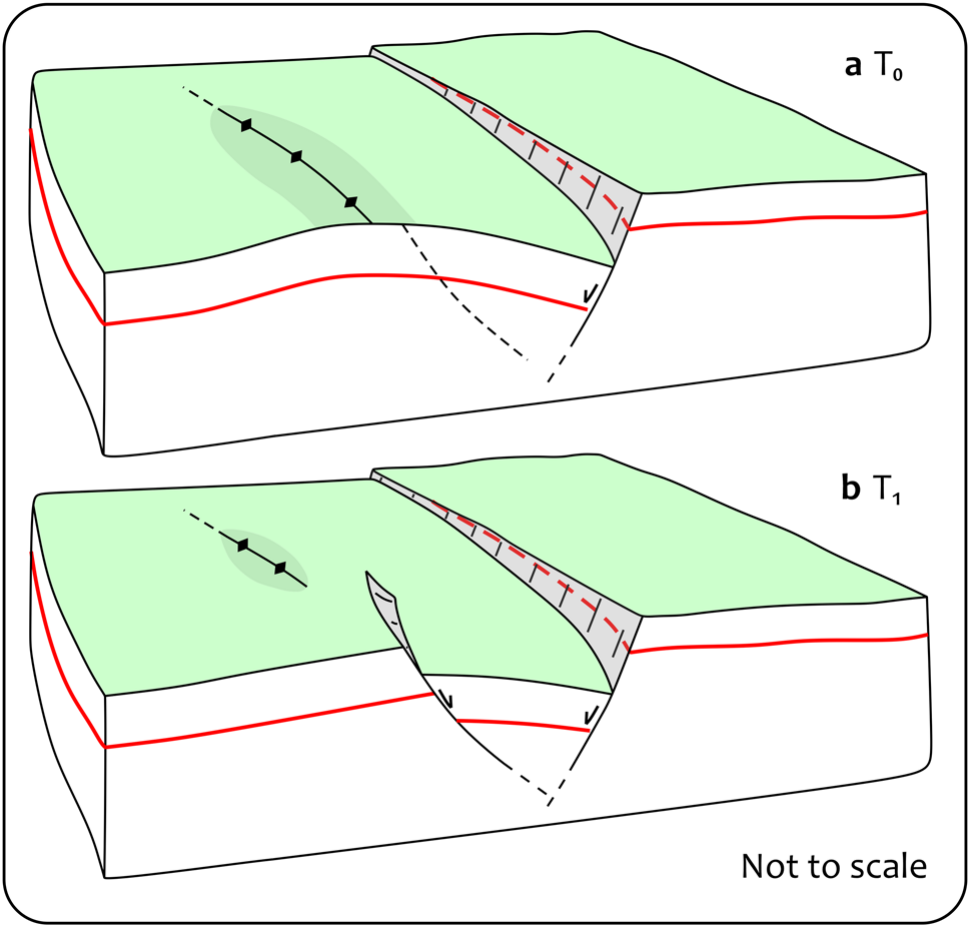
Off-fault deformation development. Conceptual model of the long wavelength hangingwall deformation. (**a**) Hangingwall bending increases where the fault displacement is higher. (**b**) Rupture of the hangingwall following its forward migration, and development of an antithetic fault.

Our results also find evidence of large-scale deformation, which is challenging to measure in the field. The clearest evidence is represented by the flexural bending of the VFS hanging wall as reconstructed in detail from our cross-sections (Figs. 3, S3 and S4). To the north, the hangingwall is downthrow and reaches a maximum subsidence of ~ 30 cm. Southward, the deformation profiles show a typical curvature, characterized by bending toward the main fault and a weak bulge, up to ~ 15–20 cm along its hinge zone. Further to the south, the deformation gradient increases and an antithetic fault forms, leading to the loss of the curvature and of the back bulge (Fig. 5). In this model, a first stage of deformation is characterized by a downthrow movement of the hangingwall following a gradual curvature toward the main fault. When the hangingwall experiences further bending, accompanied by a forward migration of the fold hinge, the strength limit of the rocks is reached, and an antithetic fault is produced to accommodate the on-fault movement.

We remark that the retrieved results provide new important constraints that can be exploited for further studies on mechanical and kinematic modelling.

## Conclusions

The new approach of DInSAR data interpretation propose in this work allows us to quickly generate a detailed map of the surface deformation patterns related to the fault segments activated during the October 2016 earthquakes in central Italy. In addition, we give insights on debated cross-cutting relationships (i.e., the southern segment of the VFS cross-cutting the MSt) as well as on large-scale deformation associated with faulting (i.e., the bending of the hanging-wall block).

Our approach does not intend to substitute fieldwork activities, but it aims to demonstrate the potential of DInSAR technique to support field survey overcoming its limitations (i.e., remote areas or ruptures hidden by alluvium deposits). In addition, the quick identification of coseismic ruptures just after major seismic events, would allow to reduce the costs and time spent in the field by efficiently driving the geologists onsite. On the other hand, the intrinsic limitations of the DInSAR technique make the fieldwork essential to identify and quantify the actual amount of coseismic displacement accrued by single ruptures and faults.

Accordingly, we suggest that future approaches, based on such data and methods, integrated with further geodetic and surface geological measurements, can be profitably employed to provide a faster and better characterization of active seismogenic faults.

We conclude that the methodological approach proposed in this paper can be potentially applied to worldwide earthquakes, based on their environmental characteristics, aiming to speed up and better focus any kind of post-earthquake interventions in the emergency phase.

## Supplementary Information

Supplementary Legends.

Supplementary Figure S1.

Supplementary Figure S2.

Supplementary Figure S3.

Supplementary Figure S4.

## Data Availability

We include the original ASCII file of the DInSAR data (VDM1 and HDM1), the raster file of the slope analysis of the VDM1 and the original coseismic ruptures in .kmz file in the FigShare repository at the https://doi.org/10.6084/m9.figshare.17128943.
