# Supplementary material for: Surface ruptures and off-fault deformation of the October 2016 central Italy earthquakes from DInSAR data
Source: Sci Rep. 2022 Feb 24;12:3172. doi: 10.1038/s41598-022-07068-9 (PMC8873333; doi:10.1038/s41598-022-07068-9)
Supplement: Supplementary file 1 — Supplementary Legends. [file 41598_2022_7068_MOESM1_ESM.docx]

**Fig. S1. InSAR interferograms.** Interferograms generated from ALOS-2 data pairs acquired along ascending orbits on (**a**) 24 August and 2 November 2016 and (**b**) 24 August 2016 and 6 September 2017, and along descending orbits on (**c**) 31 August and 9 November 2016 and (**d**) 31 August 2016 and 24 May 2017.

**Fig. S2. Displacement maps.** (**a**) Vertical (VDM1) and (**b**) East-West (HDM1) displacement maps computed by combining the ascending and descending ALOS-2 measurements acquired on 24 August and 2 November 2016, and on 31 August and 9 November 2016, respectively. (**c**) Vertical (VDM2) and (**d**) East-West (HDM2) displacement maps computed by combining the ascending and descending ALOS-2 measurements acquired on 24 August and 6 September 2017, and on 31 August and 24 May 2017, respectively. All the reported maps include the surface deformations generated by both the M_w_ 5.9 and the M_w_ 6.5 2016 October earthquakes.

**Fig. S3. VDM1 and VDM2 cross sections.**  **a, b**) VDM1 and VDM2, respectively. **c-i**) Representative cross-sections comparing the vertical displacement (VD) from the VDM1 (in black) and the VDM2 (in green), the topography (in red) and the zone affected by deformation close to the VFS (in grey).

**Fig. S4. VDM and HDM cross sections.** 3D visualization along the seven cross-sections identified in Fig.1b of the vertical (VD, in black) and horizontal (HD, in blue) displacements computed from the VDM1 and HDM1, respectively.
