# Supplementary figures and images for: Surface ruptures and off-fault deformation of the October 2016 central Italy earthquakes from DInSAR data
Source: Sci Rep. 2022 Feb 24;12:3172. doi: 10.1038/s41598-022-07068-9 (PMC8873333; doi:10.1038/s41598-022-07068-9)

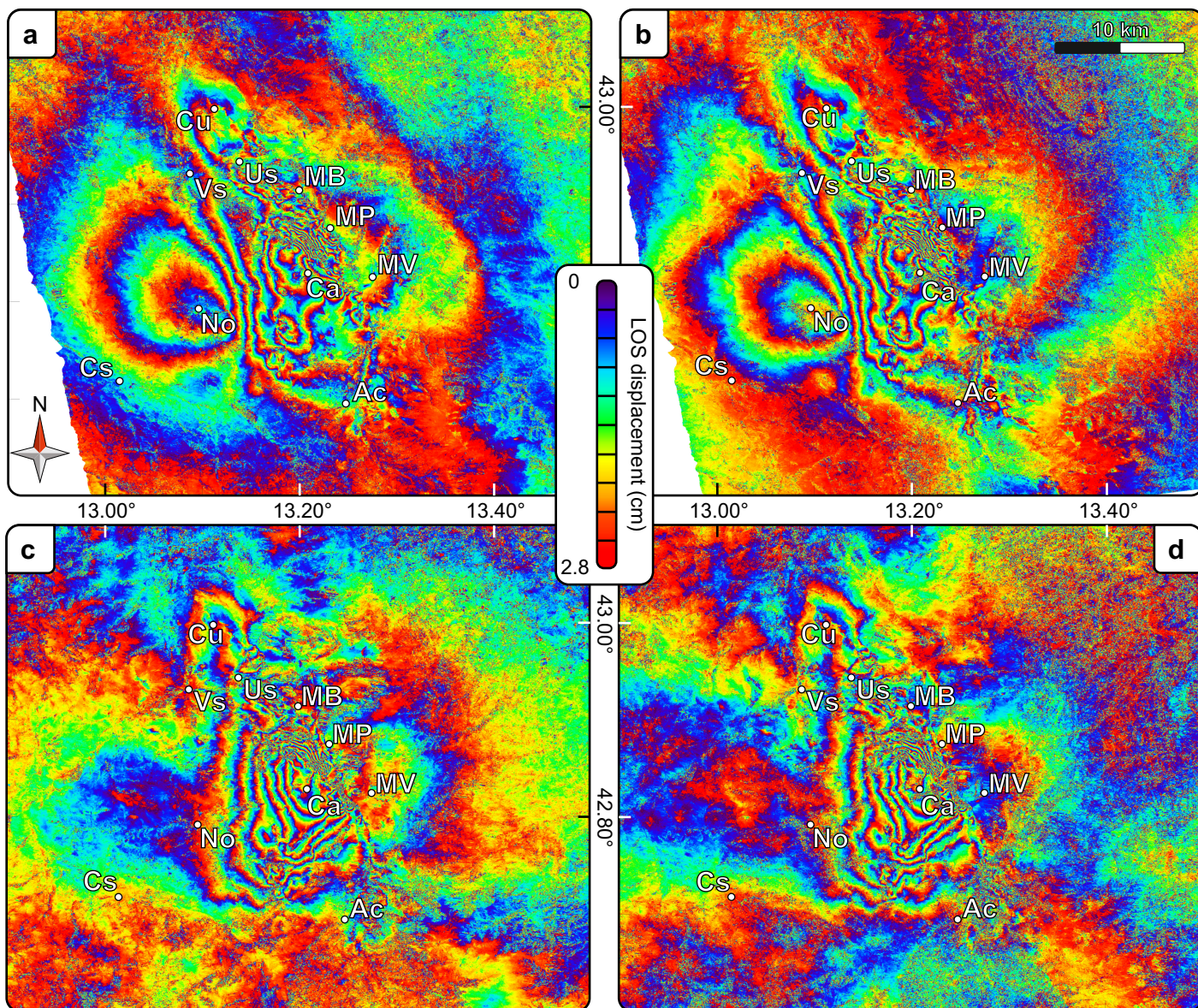

Supplement: Supplementary file 2 — Supplementary Figure S1. [file 41598_2022_7068_MOESM2_ESM.pdf]

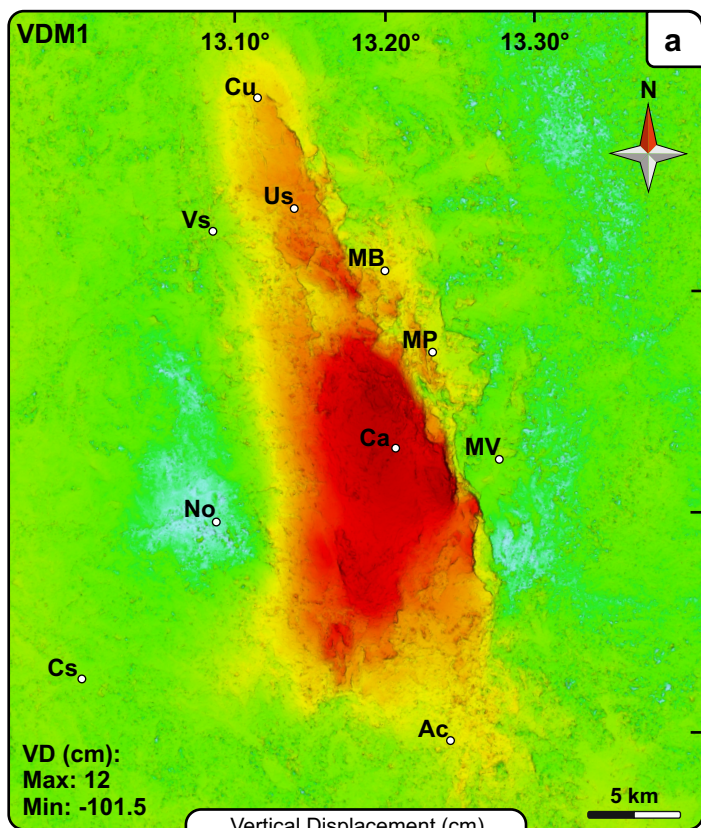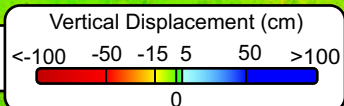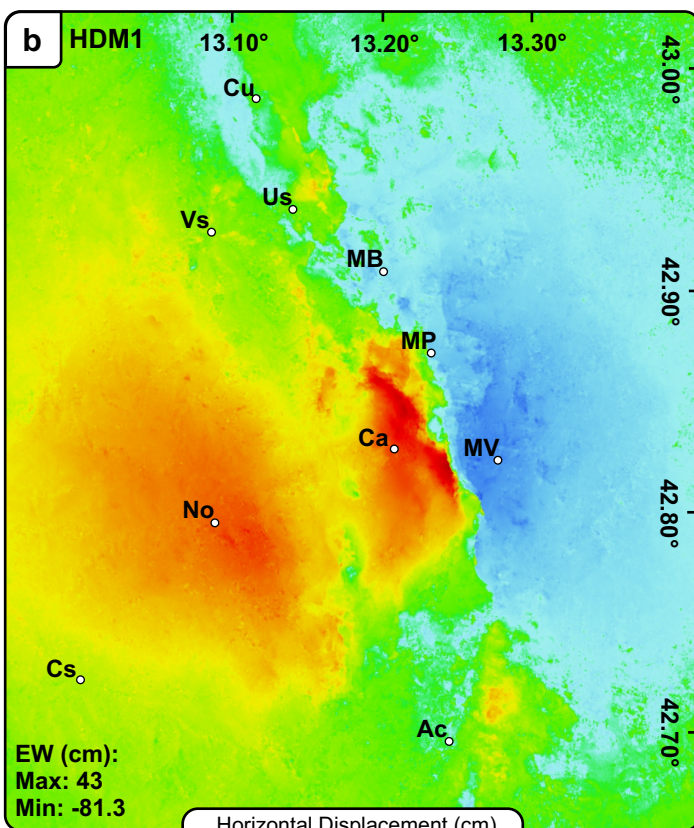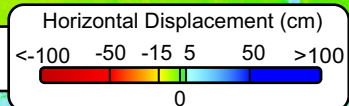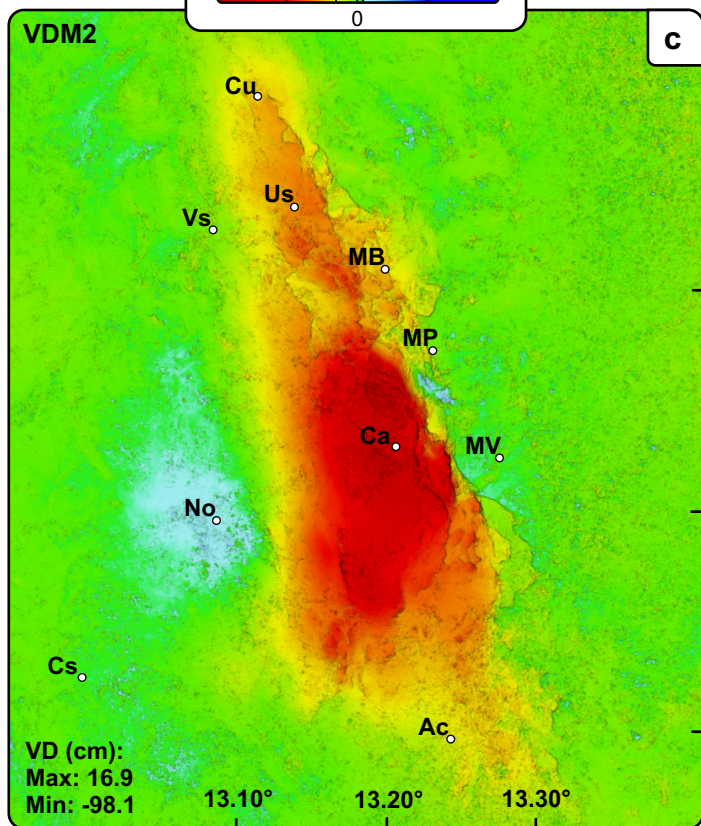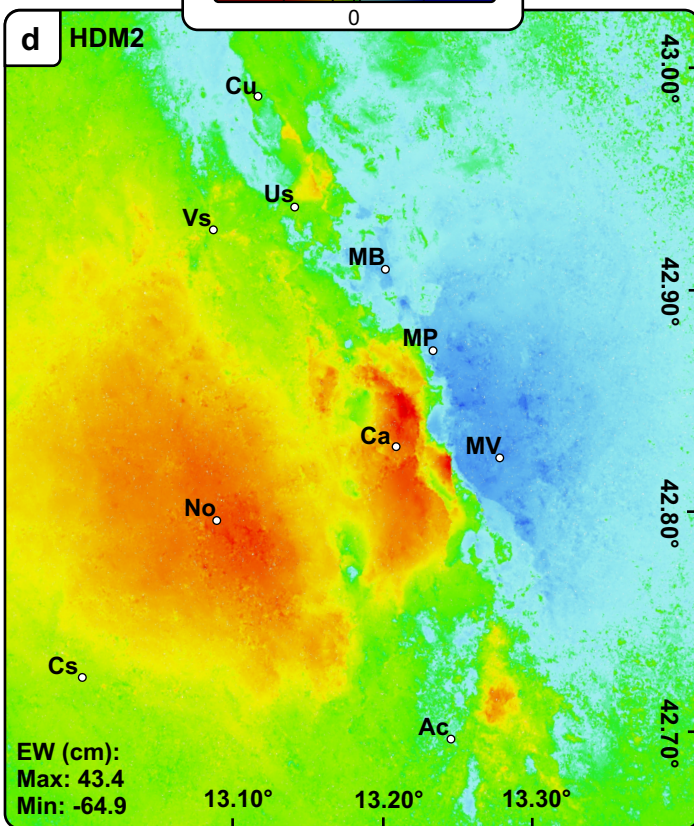

Supplement: Supplementary file 3 — Supplementary Figure S2. [file 41598_2022_7068_MOESM3_ESM.pdf]

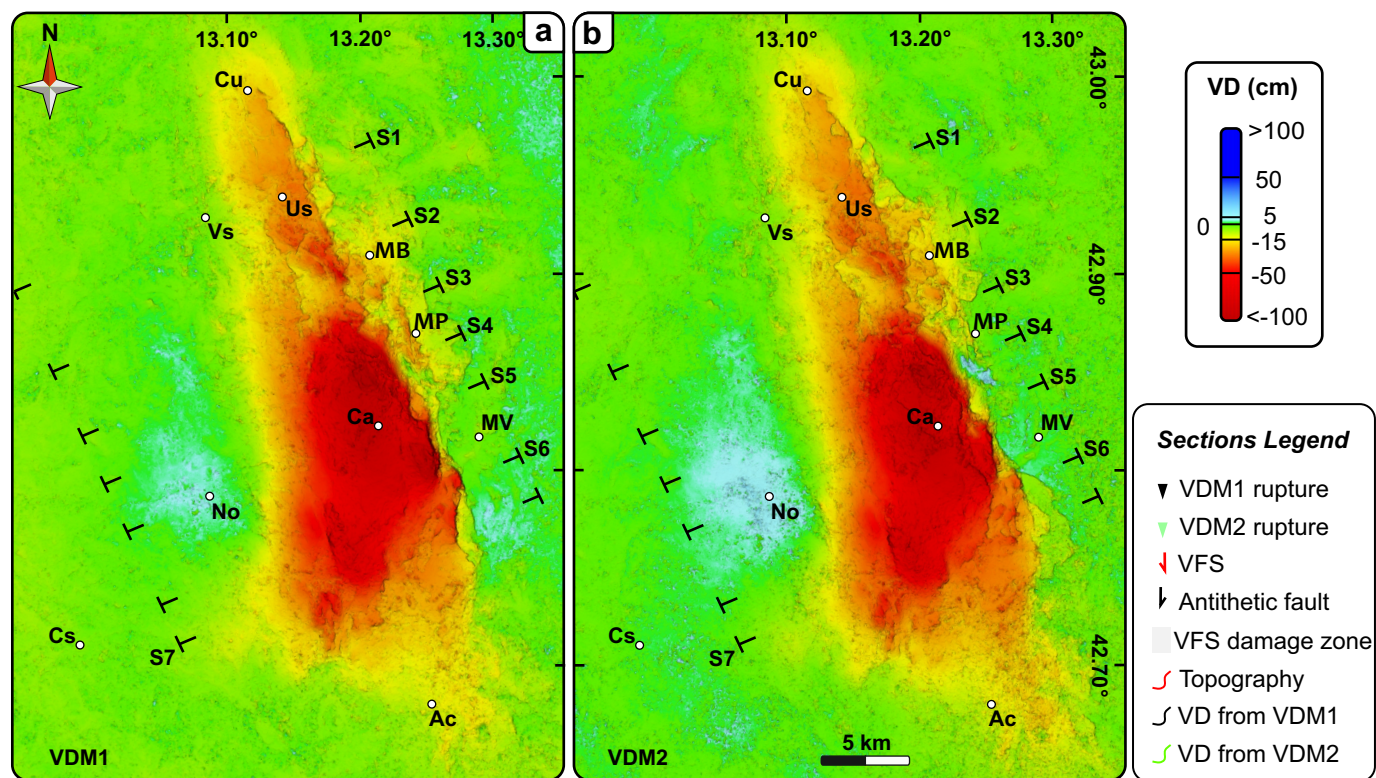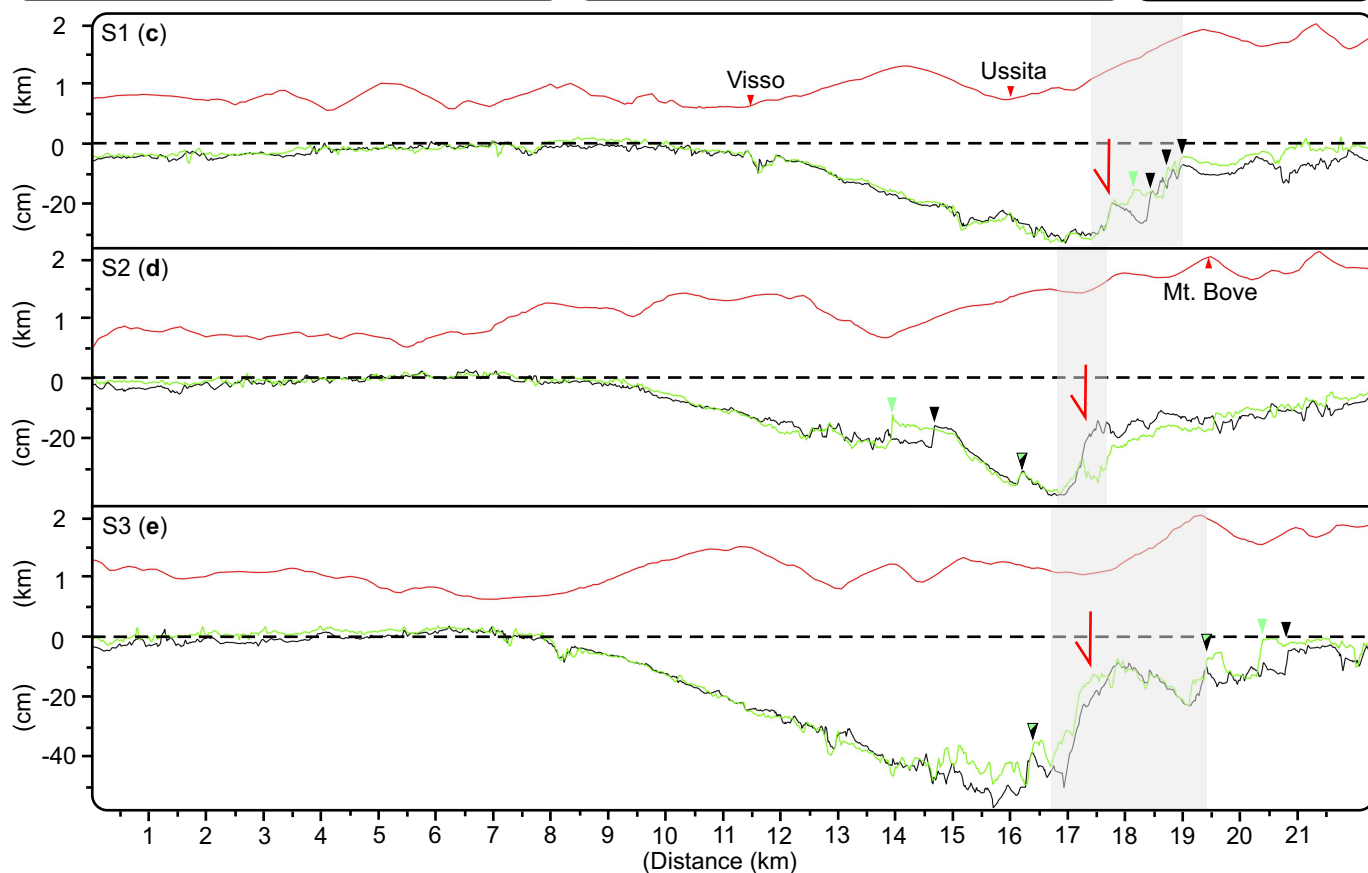

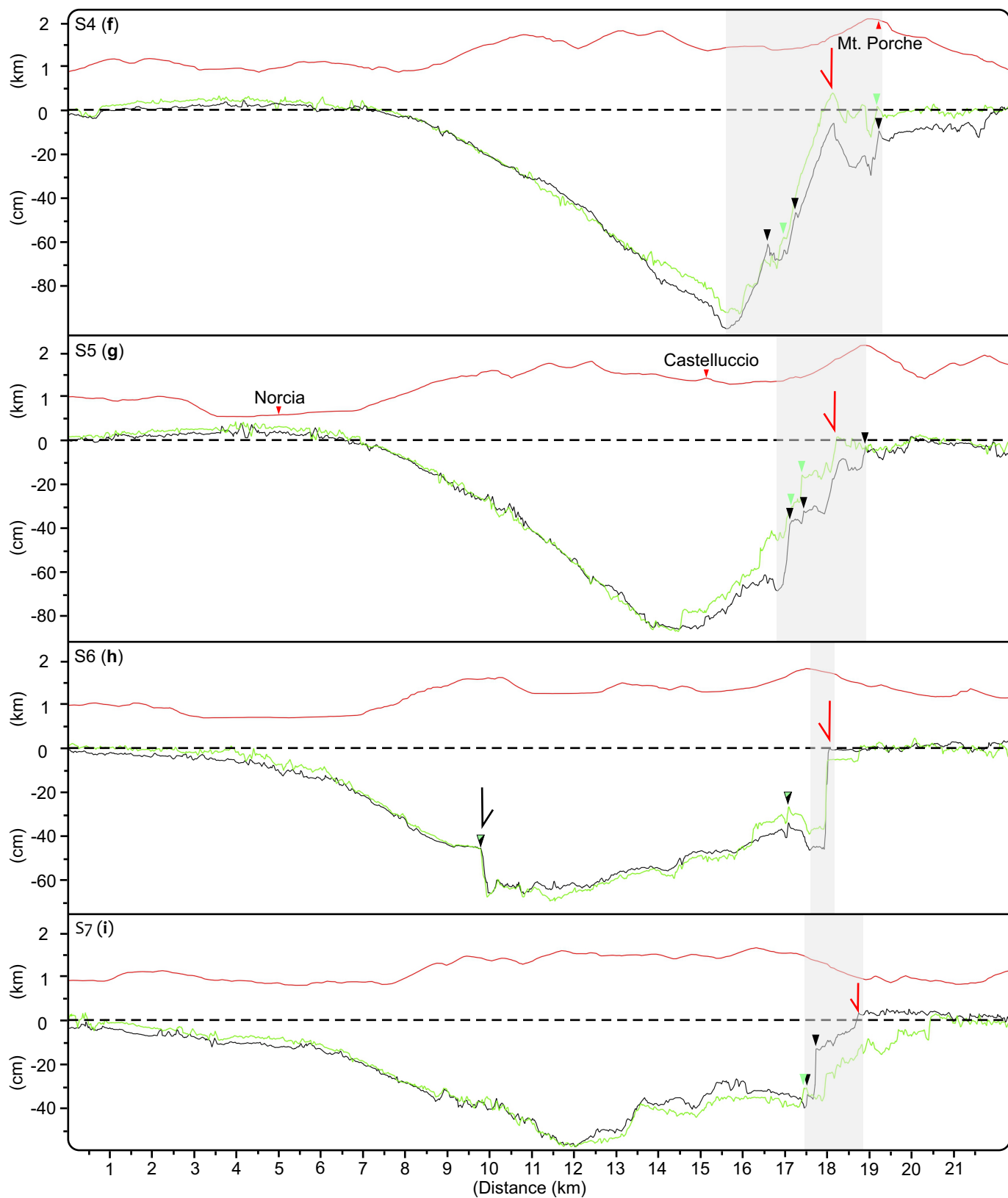

Supplement: Supplementary file 4 — Supplementary Figure S3. [file 41598_2022_7068_MOESM4_ESM.pdf]

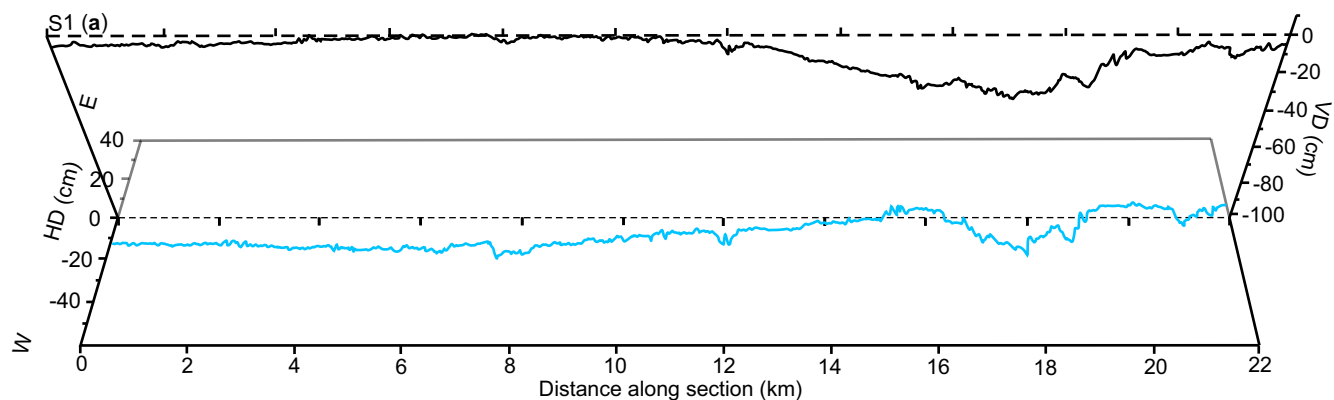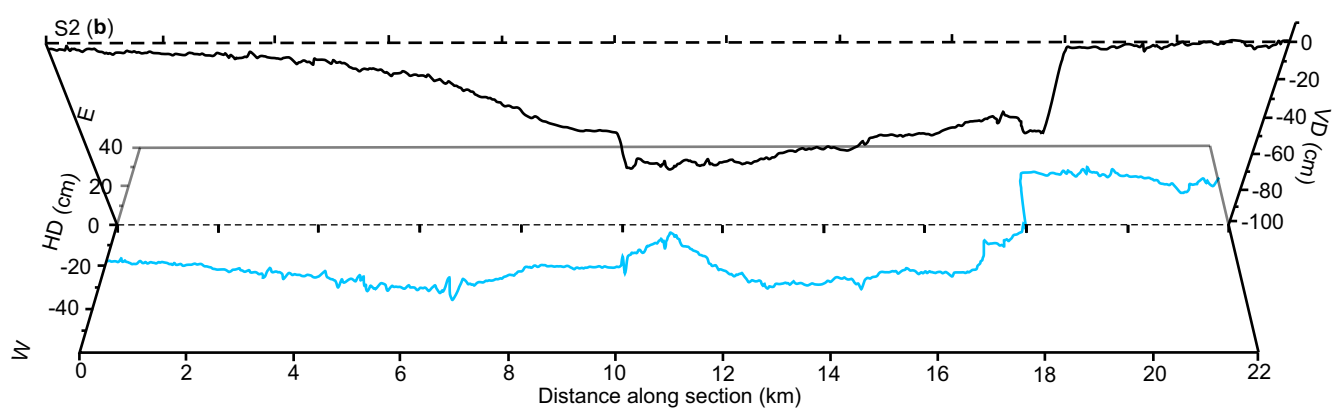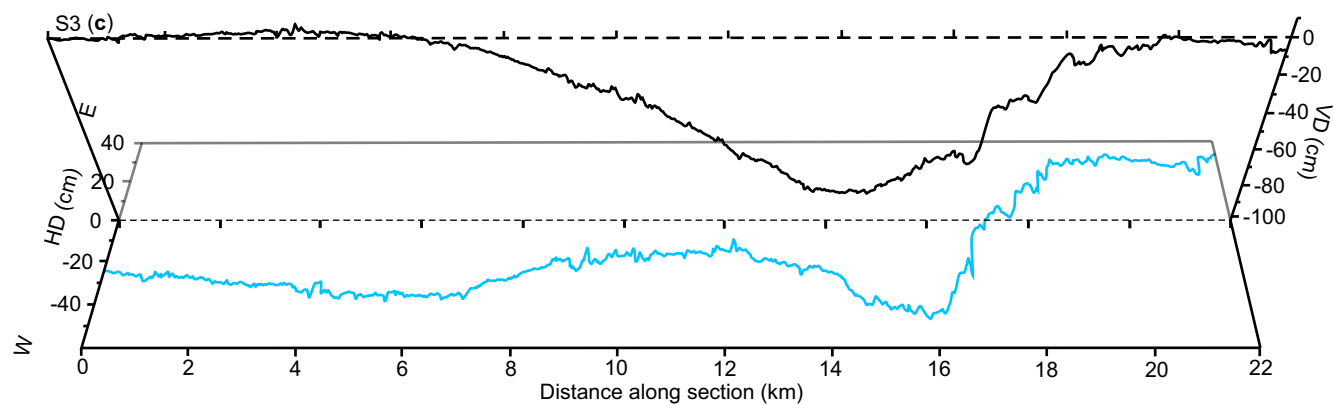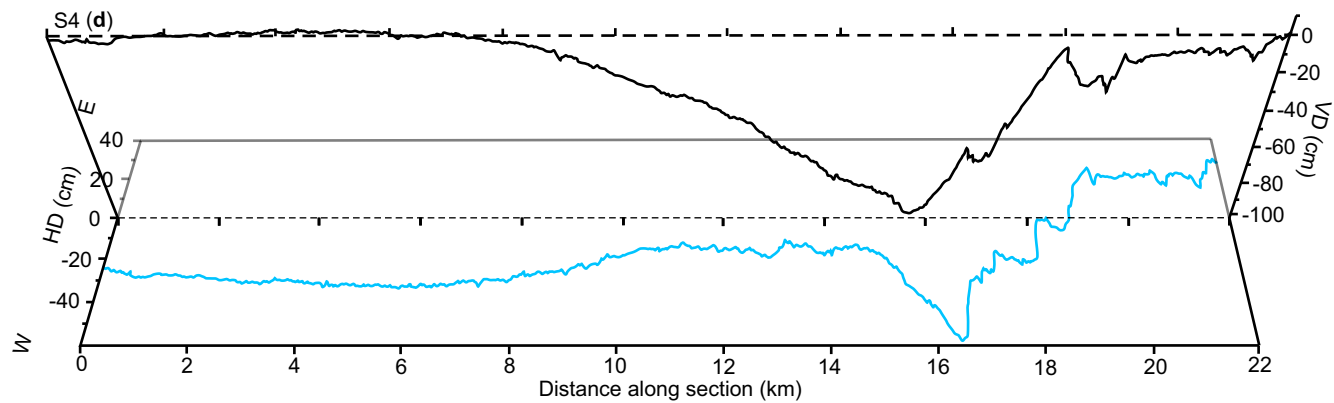

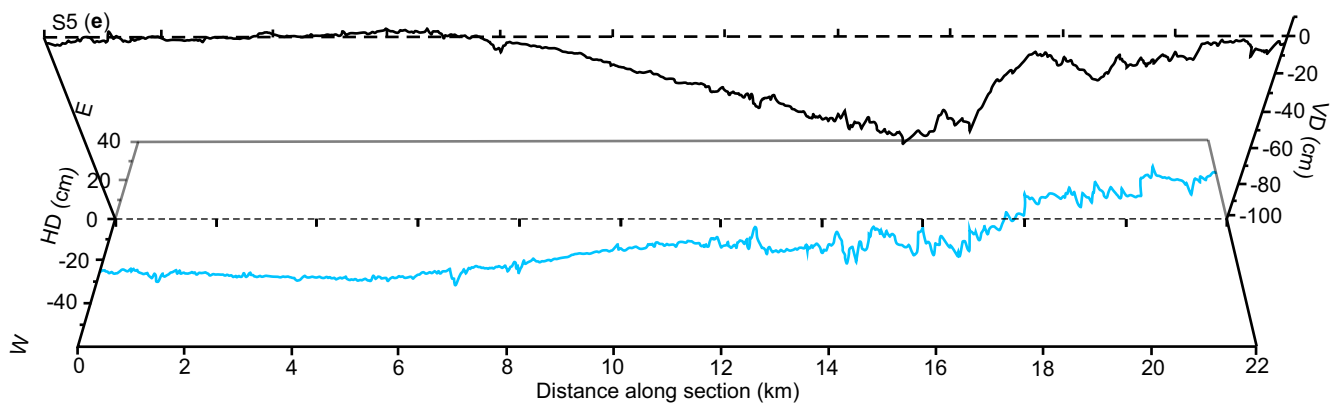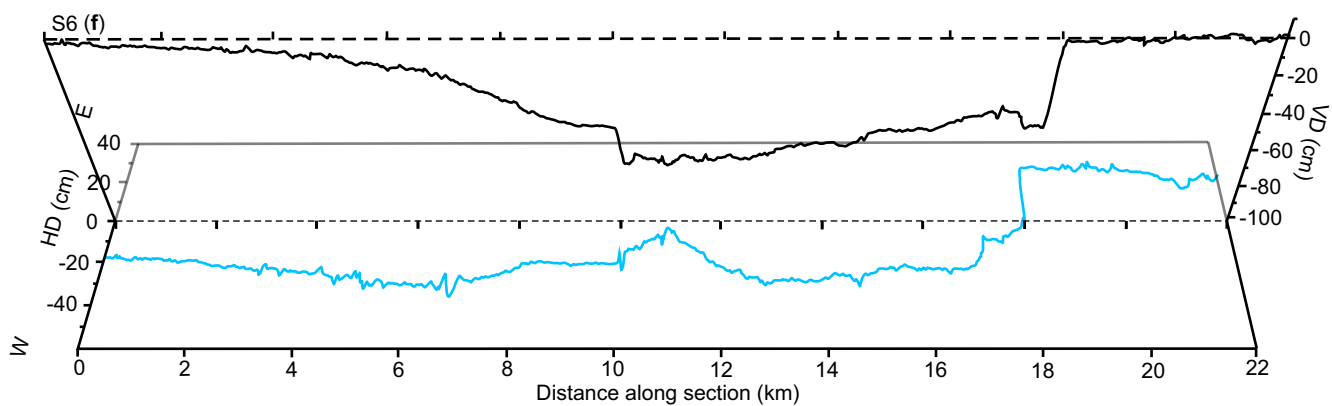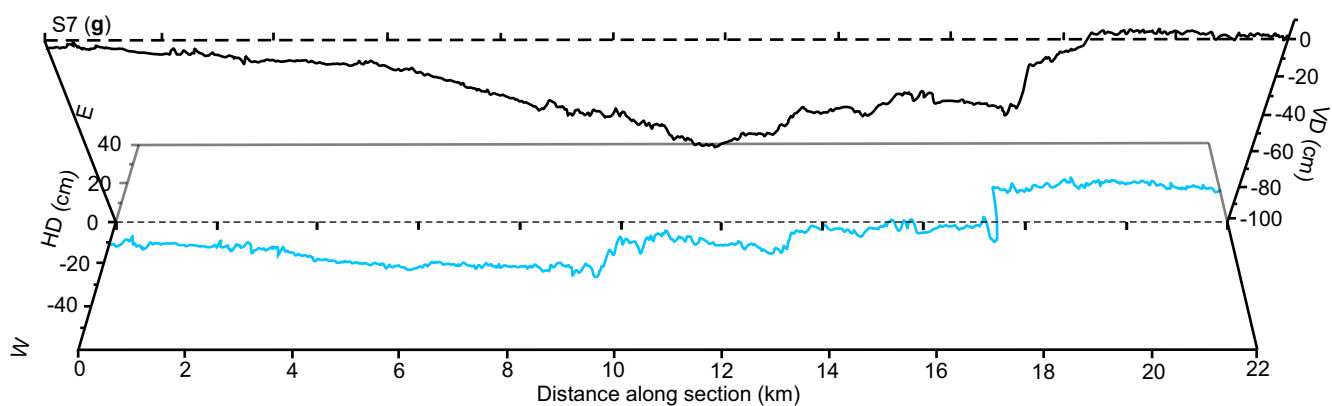

Supplement: Supplementary file 5 — Supplementary Figure S4. [file 41598_2022_7068_MOESM5_ESM.pdf]
